# (-)-Epigallocatechin Gallate Reduces Platelet-Derived Growth Factor-BB-Stimulated Interleukin-6 Synthesis in Osteoblasts: Suppression of SAPK/JNK

**DOI:** 10.1155/2008/291808

**Published:** 2009-01-12

**Authors:** Shinji Takai, Rie Matsushima-Nishiwaki, Seiji Adachi, Hideo Natsume, Chiho Minamitani, Jun Mizutani, Takanobu Otsuka, Haruhiko Tokuda, Osamu Kozawa

**Affiliations:** ^1^Department of Pharmacology, Gifu University Graduate School of Medicine, Gifu 501-1194, Japan; ^2^Department of Orthopedic Surgery, Nagoya City University Graduate School of Medical Sciences, Nagoya 467-8601, Japan; ^3^Department of Clinical Laboratory, National Hospital for Geriatric Medicine, National Center for Geriatrics and Gerontology, Obu 474-8511, Japan

## Abstract

We previously showed that the mitogen-activated protein (MAP) kinase superfamily, p44/p42 MAP kinase, p38 MAP kinase, and stress-activated protein kinase (SAPK)/c-Jun *N*-terminal (JNK), positively plays a part in the platelet-derived growth factor-BB- (PDGF-BB-) stimulated synthesis of interleukin-6 (IL-6), a potent bone resorptive agent, in osteoblast-like MC3T3-E1 cells while Akt and p70 S6 kinase negatively regulates the synthesis. In the present study, we investigated whether (-)-epigallocatechin gallate (EGCG), one of the major green tea flavonoids, affects the synthesis of IL-6 in these cells and the mechanism. EGCG significantly reduced the IL-6 synthesis and IL-6 mRNA expression stimulated by PDGF-BB, EGCG reduced the PDGF-BB-stimulated IL-6 synthesis also in primary-cultured osteoblasts. EGCG had no effect on the levels of osteocalcin and osteoprotegerin in MC3T3-E1 cells. The PDGF-BB-induced autophosphorylation of PDGF receptor *β* was not suppressed by EGCG. The PDGF-BB-induced phosphorylation of p44/p42 MAP kinase and p38 MAP kinase was not affected by EGCG. On the other hand, EGCG markedly suppressed the PDGF-BB-induced phosphorylation of SAPK/JNK. Finally, the PDGF-BB-induced phosphorylation of Akt and p70 S6 kinase was not affected by EGCG. These results strongly suggest that EGCG inhibits the PDGF-BB-stimulated synthesis of IL-6 via suppression of SAPK/JNK pathway in osteoblasts.

## 1. INTRODUCTION

Interleukin-6 (IL-6) is a multifunctional cytokine that has important physiological effects on a wide range of functions such as promoting B-cell differentiation, T-cell activation, and inducing acute phase proteins [1–4]. It is generally recognized that two functional cells, osteoblasts and osteoclasts, strictly regulate bone metabolism, the former responsible for bone formation and the latter for bone resorption [5]. The formation of bone structures and bone remodeling results from the coupling process; bone resorption by activated osteoclasts with subsequent deposition of new matrix by osteoblasts. In bone metabolism, it is well recognized that IL-6 is one of the most potent osteoclastogenic factors [3, 4]. Bone resorption is mediated by the increased local production of inflammatory cytokines such as tumor necrosis factor-*α* and IL-1. In osteoblasts [6–8], it has been reported that bone resorptive agents such as tumor necrosis factor-*α* and IL-1 stimulate the synthesis of IL-6. As for bone metabolism, IL-6 has been shown to stimulate bone resorption and induce osteoclast formation [3, 4, 7, 9]. Thus, accumulating evidence indicates that IL-6 secreted from osteoblasts plays a key role as a downstream effector of bone resorptive agents. It has been shown that platelet-derived growth factor-BB (PDGF-BB), a well-known mitogenic factor, increases proliferation and inhibits the differentiation of osteoblasts [10]. PDGF-BB also enhances bone resorption by increasing the number of osteoclasts, an effect that may be secondary to an increase in the expression of IL-6 [10, 11]. Therefore, modulation of PDGF-BB effect would be a possible therapeutic target of osteoporosis. In our recent studies [12, 13], we have reported that PDGF-BB stimulates IL-6 synthesis through p44/p42 MAP kinase, p38 MAP kinase, and stress-activated protein kinase (SAPK)/c-Jun *N*-terminal (JNK), members of the MAP kinase superfamily [14], in osteoblast-like MC3T3-E1 cells and that Akt and p70 S6 kinase limits the synthesis. However, the exact mechanism of PDGF-BB underlying the IL-6 synthesis in osteoblasts has not yet been elucidated.

Compounds in foods such as vegetables and fruits have beneficial properties to human being. Among them, it has been reported that flavonoids possess antioxidative, antibacterial, and antitumor effects [15, 16]. Catechins are one of the major flavonoids, which are present in various species of plants such as green tea [16]. In bone metabolism, it has been shown that catechin suppresses bone resorption [17]. As for osteoclasts, it has been reported that (−)-epigallocatechin gallate (EGCG) induces osteoclast apoptosis [18, 19] and suppresses the differentiation [20]. However, the effects of EGCG on the expression of cytokines or matrix-degrading enzyme from osteoclast-stimulated or treated osteoblastic cells have not been reported as far as we know. As for osteoblasts, it has been shown that catechin stimulates alkaline phosphatase activity, a mature osteoblast phenotype [5], and reduces apoptosis in osteoblast-like MC3T3-E1 cells [21]. However, the exact mechanism of catechin in osteoblasts is not fully known.

In the present study, we investigated whether (−)-epigallocatechin gallate (EGCG), one of the major green tea flavonoids, affects the PDGF-BB-stimulated IL-6 synthesis in osteoblast-like MC3T3-E1 cells and the mechanism behind it. We here show that EGCG reduces the PDGF-BB-stimulated IL-6 synthesis via attenuation of SAPK/JNK pathway in these cells.

## 2. MATERIALS AND METHODS

### 2.1. Materials

Recombinant PDGF-BB, IL-6 ELISA, osteocalcin ELISA, and osteoprotegerin (OPG) ELISA kit were purchased from R&D Systems, Inc. (Minneapolis, Minn, USA). EGCG was obtained from Calbiochem-Novabiochem Corp. (La Jolla, Calif, USA). Phospho-specific PDGF receptor *β* antibodies, PDGF receptor *β* antibodies, phospho-specific p44/p42 MAP kinase antibodies, p44/p42 MAP kinase antibodies, phospho-specific p38 MAP kinase antibodies, p38 MAP kinase antibodies, phospho-specific SAPK/JNK kinase antibodies, SAPK/JNK antibodies, phospho-specific Akt antibodies, Akt antibodies, phospho-specific p70 S6 kinase antibodies, and p70 S6 kinase antibodies were purchased from Cell Signaling Technology (Beverly, Mass, USA). ECL Western blotting detection system was purchased from Amersham Japan (Tokyo, Japan). Other materials and chemicals were obtained from commercial sources.

### 2.2. Cell culture

The cloned osteoblast-like MC3T3-E1 cells, which have been derived from newborn mouse calvaria [22], were maintained as previously described [23]. Briefly, the cells were cultured in *α*-minimum essential medium (*α*-MEM) containing 10% fetal calf serum (FCS) at 37°C in a humidified atmosphere of 5% CO_2_/95% air. The cells were seeded into 35 mm diameter dishes (5 × 10^4^/dish) or 90 mm diameter dishes (5 × 10^5^/dish) in *α*-MEM containing 10% FCS. After 5 days, the medium was exchanged for *α*-MEM containing 0.3% FCS. The cells were used for experiments after 48 hours.

Primary-cultured osteoblasts were obtained from the calvaria of newborn (1 or 2-day old) balb/c mice as previously described [24]. They were seeded into 90 mm diameter dishes (25 × 10^4^ cells) in *α*-MEM containing 10% FCS. The medium was changed every 3 days until the cells reached confluence at 5 days. Then, the medium was exchanged for *α*-MEM containing 0.3% FCS. The cells were used for experiments after 48 hours.

### 2.3. Assays for osteocalcin, OPG, and IL-6

The cultured cells were pretreated with vehicle or various doses of EGCG (1 to 30 *μ*M) for 24 hours, the levels of osteocalcin and OPG in the medium were measured by respective ELISA kit. The cultured cells were stimulated by PDGF-BB in 1 mL of *α*-MEM containing 0.3% FCS, and then incubated for the indicated periods. The conditioned medium was collected, and IL-6 in the medium was then measured by IL-6 ELISA kit. When indicated, the cells were pretreated with various doses of EGCG for 60 minutes.

### 2.4. Real-time RT-PCR

The cultured cells were pretreated with 30 *μ*M EGCG or vehicle for 60 minutes, and then stimulated by 50 ng/mL PDGF-BB for 60 minutes. Total RNA was isolated and transcribed into complementary DNA using Trizol reagent and Omniscript Reverse Transcriptase Kit. Real-time RT-PCR was performed using a Light Cycler system (Roche Diagnostics Basel, Switzerland) in capillaries and FastStart DNA Master SYBR Green I provided with the kit. Sense and antisense primers were synthesized based on the report of Simpson et al. for mouse GAPDH mRNA [25]. Sense and antisense primers for mouse IL-6 mRNA were purchased from Takara Bio Inc. (Tokyo, Japan) (primer set ID:MA039013). The amplified products were determined by melting curve analysis and agarose electrophoresis. IL-6 mRNA levels were normalized with those of GAPDH mRNA.

### 2.5. Analysis of western blotting

The cultured cells were stimulated by PDGF-BB in *α*-MEM containing 0.3% FCS for the indicated periods. The cells were washed twice with phosphate-buffered saline and then lysed, homogenized, and sonicated in a lysis buffer containing 62.5 mM Tris/HCl, pH 6.8, 2% sodium dodecyl sulfate (SDS), 50 mM dithiothreitol, and 10% glycerol. The cytosolic fraction was collected as a supernatant after centrifugation at 125, 000×g for 10 minutes at 4°C. SDS-polyacrylamide gel electrophoresis (PAGE) was performed by Laemmli [26] in 10% polyacrylamide gel. Western blotting analysis was performed as described previously [27] by using phospho-specific PDGF receptor *β* antibodies, PDGF receptor *β* antibodies, phospho-specific p44/p42 MAP kinase antibodies, p44/p42 MAP kinase antibodies, phospho-specific p38 MAP kinase antibodies, p38 MAP kinase antibodies, phospho-specific SAPK/JNK kinase antibodies, SAPK/JNK antibodies, phospho-specific Akt antibodies, Akt antibodies, phospho-specific p70 S6 kinase antibodies, and p70 S6 kinase antibodies with peroxidase-labeled antibodies raised in goat against rabbit IgG being used as second antibodies. Peroxidase activity on PVDG membrane was visualized on X-ray film by means of the ECL Western blotting detection system. When indicated, the cells were pretreated with various doses of EGCG for 60 minutes.

### 2.6. Determinations

The absorbance of enzyme immunoassay samples was measured at 450 nm with EL 340 Bio Kinetic Reader (Bio-Tek Instruments, Inc., Winooski, Vt, USA). The densitometric analysis was performed using Molecular Analyst/Macintosh (Bio-Rad Laboratories, Hercules, Calif, USA).

### 2.7. Statistical analysis

The data were analyzed by ANOVA followed by the Bonferroni method for multiple comparisons between pairs, and a *P* < .05 was considered significant. All data are presented as the mean ± SEM of triplicate determinations. Each experiment was repeated three times with similar results.

## 3. RESULTS

### 3.1. Effects of EGCG on the PDGF-BB-stimulated IL-6 synthesis in MC3T3-cells and primary-cultured mouse osteoblasts

**Figure 1:**
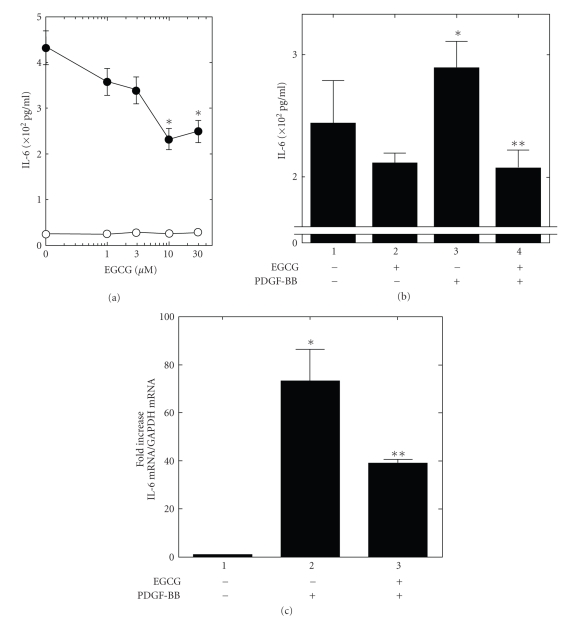
Effect of EGCG on the PDGF-BB-stimulated IL-6 synthesis and expression levels of IL-6 mRNA in osteoblasts. (a) Osteoblast-like MC3T3-E1 cells were pretreated with various doses of EGCG for 60 minutes, and then stimulated by 50 ng/mL PDGF-BB or vehicle for 24 hours. Each value represents the mean ± SEM of triplicate determinations. Similar results were obtained with two additional and different cell preparations. *P < .05: compared to the value of control. **P < .05: compared to the value of PDGF-BB alone. (b) Primary cultures of osteoblast were pretreated with or without 30 *μ*M of EGCG for 60 minutes, and then stimulated by 50 ng/mL PDGF-BB or vehicle for 24 hours. Each value represents the mean ± SEM of triplicate determinations. Similar results were obtained with two additional and different cell preparations. *P < .05: compared to value of the PDGF-BB alone. (c) Osteoblast-like MC3T3-E1 cells were pretreated with 30 *μ*M EGCG or vehicle for 60 minutes, and then stimulated by 50 ng/mL PDGF-BB for 60 minutes. Total RNA was isolated and transcribed into complementary DNA. The expressions of IL-6 mRNA and GAPDH mRNA were quantified by real-time RT-PCR. IL-6 mRNA levels were normalized with those of GAPDH mRNA. Results were standardized for the value of control (without EGCG and PDGF-BB). Each value represents the mean ± SEM of triplicate determinations. Similar results were obtained with two additional and different cell preparations. *P < .05: compared to the value of control. **P < .05: compared to the value of PDGF-BB alone.

It has been reported that PDGF-BB induces transcription of IL-6 in rat osteoblasts [11]. We have previously found that PDGF-BB stimulates IL-6 synthesis in mouse osteoblast-like MC3T3-E1 cells [12]. We first examined the effect of EGCG on the PDGF-BB-stimulated IL-6 synthesis. EGCG, which alone had little effect on the IL-6 levels, significantly reduced the PDGF-BB-stimulated synthesis of IL-6 (Figure 1(a)). EGCG (30 *μ*M) caused about 50% reduction in the PDGF-BB effect. In addition, we also examined the effect of EGCG in primary-cultured mouse osteoblasts. PDGF-BB significantly enhanced IL-6 synthesis in primary osteoblasts (Figure 1(b)). Furthermore, EGCG (30 *μ*M) significantly reduced the PDBF-BB-stimulated synthesis of IL-6 (Figure 1(b)). We next assessed the effect of EGCG on cell viability by trypan blue dye exclusion test. We confirmed that the viability of MC3T3-E1 cells incubated at 37°C for 24 hours in the presence of 30 *μ*M EGCG was more than 90% in comparison to that of the control cells. To determine whether EGCG could affect the cell proliferation, we counted the cell number before and after the 24-hour incubation with 30 *μ*M EGCG. We confirmed that EGCG did not affect the cell number at a dose of 30 *μ*M (9.7 ± 1.1 × 10^5^ cells/mL for control; 9.1 ± 1.6 × 10^5^ cells/mL for 30 *μ*M EGCG as measured during the stimulation for 24 hours). Therefore, EGCG at 30 *μ*M hardly affects the cell viability or proliferation of osteoblast-like MC3T3-E1 cells up to 24 hours.

### 3.2. Effects of EGCG on the levels of osteocalcin and osteoprotegerin in MC3T3-E1-cells

Next, to determine whether EGCG affects the differentiation of these cells, we examined the effect of EGCG on the synthesis of osteocalcin, a mature osteoblast phenotype [28], and osteoprotegerin (OPG), produced by osteoblasts and inhibiting osteoclastic bone resorption [29], synthesis in MC3T3-E1 cells. We found that EGCG had no effect on the osteocalcin (not detectable under the experimental condition at all; <1.56 ng/mL) and OPG (1967 ± 34 pg/mL for vehicle; 1846 ± 46 pg/mL with 30 *μ*M of EGCG) synthesis.

### 3.3. Effect of EGCG on the PDGF-BB-induced expression levels of IL-6 mRNA in MC3T3-E1 cells

To clarify whether the suppressive effect by EGCG of PDGF-BB-stimulated IL-6 synthesis is mediated through transcriptional event or not, we examined the effect of EGCG on the PDGF-BB-induced IL-6 mRNA expression by real-time RT-PCR. We found that EGCG (30 *μ*M) significantly downregulated the IL-6 mRNA expression levels 60 minutes after the stimulation (Figure 1(c)), suggesting that the suppressive effect of EGCG is mediated at least in part by the reduction of IL-6 synthesis in osteoblast-like MC3T3-E1 cells.

### 3.4.Effect of EGCG on the PDGF-BB-induced autophosphorylation of PDGF receptor *β* in MC3T3-E1 cells

**Figure 2:**
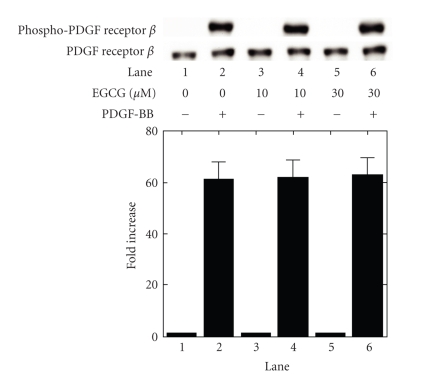
Effect of EGCG on the PDGF-BB-induced autophosphorylation of PDGF receptor *β* in MC3T3-E1 cells. The cultured cells were pretreated with the indicated doses of EGCG or vehicle for 60 minutes, and then stimulated by 50 ng/mL PDGF-BB or vehicle for 3 minutes. The extracts of cells were subjected to SDS-PAGE with subsequent Western blotting analysis with antibodies against phospho-specific PDGF receptor *β* or PDGF receptor *β*. The histogram shows quantitative representations of the levels of PDGF-BB-induced autophosphorylation obtained from laser densitometric analysis of three independent experiments. Each value represents the mean ± SEM of triplicate determinations. Similar results were obtained with two additional and different cell preparations.

In order to clarify the inhibitory mechanism of EGCG behind the PDGF-BB-stimulated IL-6 synthesis in these cells, we next examined the effect of EGCG on the PDGF-BB-induced autophosphorylation of PDGF receptor *β*. EGCG failed to affect the PDGF-BB-induced autophosphorylation of PDGF receptor *β* (Figure 2). These results lead us to speculate that the mechanism of EGCG-effect on the PDGF-BB-stimulated IL-6 synthesis may be mediated through downstream of PDGF receptor activation.

### 3.5. Effects of EGCG on the PDGF-BB-induced phosphorylation of p44/p42 MAP kinase, p38 MAP kinase, or SAPK/JNK in MC3T3-E1 cells

**Figure 3:**
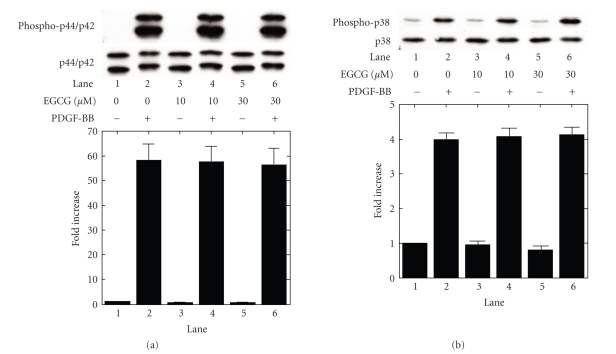
Effect of EGCG on the phosphorylation of p44/p42 MAP kinase and p38 MAP kinase induced by PDGF-BB in MC3T3-E1 cells. The cultured cells were pretreated with the indicated doses of EGCG or vehicle for 60 minutes, and then stimulated by 50 ng/mL PDGF-BB or vehicle for 3 minutes. The extracts of cells were subjected to SDS-PAGE with subsequent Western blotting analysis with antibodies against (a) phospho-specific p44/p42 MAP kinase or p44/p42 MAP kinase, and (b) phospho-specific p38 MAP kinase or p38 MAP kinase. The histogram shows quantitative representations of the levels of PDGF-BB-induced phosphorylation obtained from laser densitometric analysis of three independent experiments. Each value represents the mean ± SEM of triplicate determinations. Similar results were obtained with two additional and different cell preparations.

**Figure 4:**
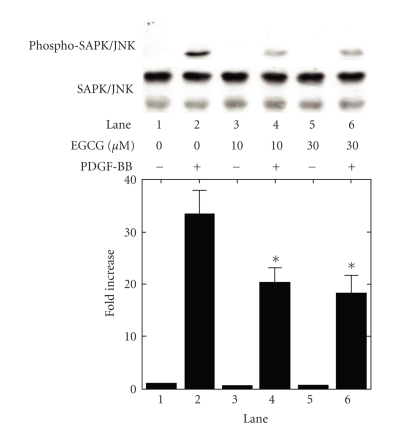
Effect of EGCG on the phosphorylation of SAPK/JNK induced by PDGF-BB in MC3T3-E1 cells. The cultured cells were pretreated with the indicated doses of EGCG or vehicle for 60 minutes, and then stimulated by 50 ng/mL PDGF-BB or vehicle for 3 minutes. The extracts of cells were subjected to SDS-PAGE with subsequent Western blotting analysis with antibodies against phospho-specific SAPK/JNK or SAPK/JNK. The histogram shows quantitative representations of the levels of PDGF-BB-induced phosphorylation obtained from laser densitometric analysis of three independent experiments. Each value represents the mean ± SEM of triplicate determinations. Similar results were obtained with two additional and different cell preparations. *P < .05: compared to the value of PDGF-BB alone.

In our previous study [13], we reported that PDGF-BB activates three major MAP kinases, p44/p42 MAP kinase, p38 MAP kinase, and SAPK/JNK, resulting in the synthesis of IL-6 in MC3T3-E1 cells. We next examined the effect of EGCG on the PDGF-BB-induced phosphorylation of p44/p42 MAP kinase, p38 MAP kinase, or SAPK/JNK. EGCG failed to affect the PDGF-BB-induced phosphorylation of p44/p42 MAP kinase or p38 MAP kinase (Figures 3(a) and 3(b)). On the contrary, EGCG, which by itself had little effect on the phosphorylation levels of SAPK/JNK, significantly suppressed the PDGF-BB-induced SAPK/JNK phosphorylation (Figure 4). According to the densitometric analysis, EGCG (30 *μ*M) caused about 40% reduction in the PDGF-BB-effect.

### 3.6. Effects of EGCG on the PDGF-BB-induced phosphorylation of Akt or p70 S6 kinase induced by PDGF-BB in MC3T3-E1 cells

**Figure 5:**
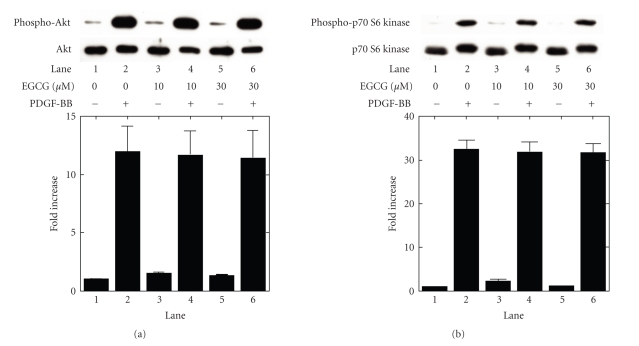
Effect of EGCG on the phosphorylation of Akt and p70 S6 kinase induced by PDGF-BB in MC3T3-E1 cells. The cultured cells were pretreated with the indicated doses of EGCG or vehicle for 60 minutes, and then stimulated by 50 ng/mL PDGF-BB or vehicle for 20 minutes. The extracts of cells were subjected to SDS-PAGE with subsequent Western blotting analysis with antibodies against (a) phospho-specific Akt or Akt, and (b) phospho-specific p70 S6 kinase or p70 S6 kinase. The histogram shows quantitative representations of the levels of PDGF-BB-induced phosphorylation obtained from laser densitometric analysis of three independent experiments. Each value represents the mean ± SEM of triplicate determinations. Similar results were obtained with two additional and different cell preparations.

In our previous studies [12, 13], we demonstrated that the PDGF-BB-activated Akt and p70 S6 kinase limit the synthesis of IL-6 in MC3T3-E1 cells. In order to investigate whether EGCG-effect on the PDGF-BB-stimulated IL-6 synthesis is mediated via the activation of Akt or p70 S6 kinase in these cells, we examined the effect of EGCG on the PDGF-BB-induced phosphorylation of Akt or p70 S6 kinase. However, EGCG had little effect on the PDGF-BB-induced phosphorylation of Akt (Figure 5(a)) or p70 S6 kinase (Figure 5(b)).

## 4. DISCUSSION

In the present study, we showed that EGCG significantly suppressed the PDGF-BB-stimulated IL-6 synthesis and expression levels of IL-6 mRNA in osteoblast-like MC3T3-E1 cells. We found that EGCG reduced the PDGF-BB-stimulated IL-6 synthesis also in primary-cultured mouse osteoblasts. These findings suggest that the inhibitory effect of EGCG on the PDGF-BB-stimulated IL-6 synthesis is not specific in a clonal osteoblast-like MC3T3-E1 cells but it is common in osteoblasts. We confirmed that EGCG at 30 *μ*M hardly affects the cell viability or proliferation of osteoblast-like MC3T3-E1 cells up to 24 hours. As for the effects of EGCG on the differentiation of these cells, the results showed that EGCG had no effect on the osteocalcin and OPG synthesis. However, it has been shown that catechin stimulates the alkaline phosphatase activity, a mature osteoblast phenotype [5], and reduces bone-resorptive cytokine production in osteoblast-like MC3T3-E1 cells [21]. Taking these results into account, it is suggested that EGCG can partially affect the differentiation of osteoblast-like MC3T3-E1 cells. We next investigated the mechanism of EGCG underlying the inhibitory effect on the IL-6 synthesis. It is well known that the MAP kinase superfamily plays a crucial role in cellular functions including proliferation, differentiation, and survival in a variety of cells [14]. Three major MAP kinases, p44/p42 MAP kinase, p38 MAP kinase, and SAPK/JNK, are known as central elements used by mammalian cells to transduce the diverse messages [14]. We have previously reported that SAPK/JNK act as positive regulators in PDGF-BB-induced IL-6 synthesis in MC3T3-E1 cells [13]. In the present study, we showed that EGCG did not affect the PDGF-BB-induced phosphorylation of p44/p42 MAP kinase or p38 MAP kinase. Therefore, it seems unlikely that EGCG reduces the PDGF-BB-stimulated IL-6 synthesis through downregulating activation of p44/p42 MAP kinase or p38 MAP kinase in osteoblast-like MC3T3-E1 cells. On the other hand, we showed that the PDGF-BB-induced phosphorylation of SAPK/JNK was markedly suppressed by EGCG. These results suggest that EGCG downregulates the PDGF-BB-stimulated activation of SAPK/JNK. Taking our findings into account, it is most likely that EGCG inhibits PDGF-BB-stimulated IL-6 synthesis through downregulating the activation of SAPK/JNK in osteoblast-like MC3T3-E1 cells. Further investigations are required to clarify the precise mechanism of catechin underlying the suppression of IL-6 synthesis in osteoblasts.

We previously showed that Akt and p70 S6 kinase negatively regulates the PDGF-BB-stimulated synthesis of IL-6 in osteoblast-like MC3T3-E1 cells [12, 13]. We additionally investigated the involvement of Akt and p70 S6 kinase in the inhibitory effect of EGCG on the IL-6 synthesis. However, EGCG failed to affect the PDGF-BB-induced phosphorylation of Akt or p70 S6 kinase. Therefore, it seems unlikely that EGCG inhibits PDGF-BB-induced IL-6 synthesis via downregulating the activation of Akt or p70 S6 kinase in osteoblast-like MC3T3-E1 cells. Taking our findings into account, it is most likely that EGCG inhibits PDGF-BB-stimulated IL-6 synthesis through downregulating the activation of SAPK/JNK in osteoblast-like MC3T3-E1 cells.

IL-6, which is synthesized from osteoblasts, modulates a variety of bone cell function [3]. In bone metabolism, IL-6 secreted from osteoblasts acts as an autocrine/paracrine factor, which induces osteoclast formation and stimulates its activity to resorb bone [4, 7]. Based on our results, it is probable that catechin-induced suppression of SAPJ/JNK activation by PDGF-BB has an inhibitory effect on bone resorption via downregulating IL-6 synthesis in osteoblasts. It has been reported that PDGF-BB is recognized as a potent stimulator of osteoblast proliferation and collagen synthesis [30]. PDGF, released during platelet aggregation, has a pivotal role in fracture healing as a systemic factor, and that PDGF also regulates bone remodeling as a local factor [30]. As for osteoporosis, one of the major problems in the health of elderly persons in advanced countries, it is reported that administration of recombinant PDGF-BB accelerates fracture healing in the geriatric, osteoporotic rat [31]. However, the present study indicates that PDBG-BB stimulates IL-6 synthesis, one of the potent bone resorptive agents, not only by osteoblast-like MC3T3-E1 cells but also by cultured primary osteoblast. In the present study, noteworthy, EGCG reduced PDGF-BB-stimulated IL-6 synthesis in osteoblasts. Therefore, our present findings led us to speculate that EGCG might enhance the fracture healing properties of PDGF-BB by reducing the IL-6 synthesis in osteoblasts. Our present data would provide a new insight in pharmacological effects of catechin on bone metabolism.

The pharmacokinetics of EGCG in human volunteers taking single dosage of 1 600 mg/day showed a rapid absorption, with a maximum plasma concentration value of 11.08 *μ*M (= 3392 ng/mL); the time to reach maximum plasma concentration was 2.2 hours, and the terminal elimination half-life ranged between 1.9 and 4.6 hours [32]. Interestingly, 10-day repeated administration of oral doses of EGCG of up to 800 mg/day was found to be safe and very well tolerated [33]. In the present study, we showed that the significant suppressive effect of EGCG on the PDGF-BB-stimulated IL-6 synthesis was clearly observed at 10 *μ*M. Therefore, it is most likely that one could drink enough green tea to reach in vivo levels which are used in our in vitro study. In addition, it has been shown that the plasma concentration of EGCG required for cancer prevention or anti-inflammatory effects is over 10 *μ*M to 50 *μ*M [34–36]. Our results, regarding suppression of IL-6 synthesis, are consistent with these previous findings. Little is known regarding the effective concentration of EGCG requiredto modulate intracellular signaling pathways. Further investigations using primary-cultured osteoblasts in addition to MC3T3-E1 cells would be necessary to elucidate the exact roles of catechin in the bone metabolism.

In conclusion, our present results strongly suggest that catechin inhibits the PDGF-BB-stimulated synthesis of IL-6 via suppression of SAPK/JNK pathway in osteoblasts.

## References

[1] Akira S, Taga T, Kishimoto T (1993). Interleukin-6 in biology and medicine. *Advances in Immunology*.

[2] Heymann D, Rousselle A-V (2000). gp130 cytokine family and bone cells. *Cytokine*.

[3] Steeve KT, Marc P, Sandrine T, Dominique H, Yannick F (2004). IL-6, RANKL, TNF-alpha/IL-1: interrelations in bone resorption pathophysiology. *Cytokine & Growth Factor Reviews*.

[4] Blair HC, Robinson LJ, Zaidi M (2005). Osteoclast signalling pathways. *Biochemical and Biophysical Research Communications*.

[5] Nijweide PJ, Burger EH, Feyen JHM (1986). Cells of bone: proliferation, differentiation, and hormonal regulation. *Physiological Reviews*.

[6] Helle M, Brakenhoff JPJ, De Groot ER, Aarden LA (1988). Interleukin 6 involved in interleukin 1-induced activities. *European Journal of Immunology*.

[7] Ishimi Y, Miyaura C, Jin CH (1990). IL-6 is produced by osteoblasts and induces bone resorption. *The Journal of Immunology*.

[8] Littlewood AJ, Russell J, Harvey GR, Hughes DE, Russell RGG, Gowen M (1991). The modulation of the expression of IL-6 and its receptor in human osteoblasts in vitro. *Endocrinology*.

[9] Roodman GD (1992). Perspectives: interleukin-6: an osteotropic factor?. *Journal of Bone and Mineral Research*.

[10] Canalis E, Marcus R (2008). Skeletal growth factors. *Osteoporosis*.

[11] Franchimon N, Durant D, Rydziel S, Canalis E (1999). Platelet-derived growth factor induces interleukin-6 transcription in osteoblasts through the activator protein-1 complex and activating transcription factor-2. *Journal of Biological Chemistry*.

[12] Hanai Y, Tokuda H, Ohta T, Matsushima-Nishiwaki R, Takai S, Kozawa O (2006). Phosphatidylinositol 3-kinase/Akt auto-regulates PDGF-BB-stimulated interleukin-6 synthesis in osteoblasts. *Journal of Cellular Biochemistry*.

[13] Takai S, Tokuda H, Hanai Y, Kozawa O (2007). Limitation by p70 S6 kinase of platelet-derived growth factor-BB-induced interleukin 6 synthesis in osteoblast-like MC3T3-E1 cells. *Metabolism*.

[14] Kyriakis JM, Avruch J (2001). Mammalian mitogen-activated protein kinase signal transduction pathways activated by stress and inflammation. *Physiological Reviews*.

[15] Jankun J, Selman SH, Swiercz R, Skrzypczak-Jankun E (1997). Why drinking green tea could prevent cancer. *Nature*.

[16] Harborne JB, Williams CA (2000). Advances in flavonoid research since 1992. *Phytochemistry*.

[17] Delaisse JM, Eeckhout Y, Vaes G (1986). Inhibition of bone resorption in culture by (+)-catechin. *Biochemical Pharmacology*.

[18] Nakagawa H, Wachi M, Woo J-T (2002). Fenton reaction is primarily involved in a mechanism of (3)-epigallocatechin-3-gallate to induce osteoclastic cell death. *Biochemical & Biophysical Research Communications*.

[19] Yun J-H, Kim C-S, Cho K-S, Chai J-K, Kim C-K, Choi S-H (2007). (-)-Epigallocatechin gallate induces apoptosis, via caspase activation, in osteoclasts differentiated from RAW 264.7 cells. *Journal of Periodontal Research*.

[20] Morinobu A, Biao W, Tanaka S (2008). (-)-Epigallocatechin-3-gallate suppresses osteoclast differentiation and ameliorates experimental arthritis in mice. *Arthritis and Rheumatism*.

[21] Choi E-M, Hwang J-K (2003). Effects of (+)-catechin on the function of osteoblastic cells. *Biological & Pharmaceutical Bulletin*.

[22] Sudo H, Kodama HA, Amagai Y, Yamamoto Y, Kasai S (1983). In vitro differentiation and calcification in a new clonal osteogenic cell line derived from newborn mouse calvaria. *Journal of Cell Biology*.

[23] Kozawa O, Suzuki A, Tokuda H, Uematsu T (1997). Prostaglandin F_2_*α*__ stimulates interleukin-6 synthesis via activation of PKC in osteoblast-like cells. *American Journal of Physiology*.

[24] Yoshida M, Niwa M, Ishisaki A (2004). Methotrexate enhances prostaglandin D2-stimulated heat shock protein 27 induction in osteoblasts. *Prostaglandins Leukotrienes and Essential Fatty Acids*.

[25] Simpson DAC, Feeney S, Boyle C, Stitt AW (2000). Retinal VEGF mRNA measured by SYBR Green I fluorescence: a versatile approach to quantitative PCR. *Molecular Vision*.

[26] Laemmli UK (1970). Cleavage of structural proteins during the assembly of the head of bacteriophage T4. *Nature*.

[27] Kato K, Ito H, Hasegawa K, Inaguma Y, Kozawa O, Asano T (1996). Modulation of the stress-induced synthesis of hsp27 and *α*B-crystallin by cyclic AMP in C6 rat glioma cells. *Journal of Neurochemistry*.

[28] Ducy P, Desbois C, Boyce B (1996). Increased bone formation in osteocalcin-deficient mice. *Nature*.

[29] Khosla S (2001). Minireview: the OPG/RANKL/RANK system. *Endocrinology*.

[30] Canalis E, Varghese S, McCarthy TL, Centrella M (1992). Role of platelet derived growth factor in bone cell function. *Growth Regulation*.

[31] Hollinger JO, Onikepe AO, MacKrell J (2008). Accelerated fracture healing in the geriatric, osteoporotic rat with recombinant human platelet-derived growth factor-BB and an injectable beta-tricalcium phosphate/collagen matrix. *Journal of Orthopaedic Research*.

[32] Ullmann U, Haller J, Decourt JP (2003). A single ascending dose study of epigallocatechin gallate in healthy volunteers. *Journal of International Medical Research*.

[33] Ullmann U, Haller J, Decourt JD, Girault J, Spitzer V, Weber P (2004). Plasma-kinetic characteristics of purified and isolated green tea catechin epigallocatechin gallate (EGCG) after 10 days repeated dosing in healthy volunteers. *International Journal for Vitamin and Nutrition Research*.

[34] Lambert JD, Yang CS (2003). Mechanisms of cancer prevention by tea constituents. *The Journal of Nutrition*.

[35] Wheeler DS, Catravas JD, Odoms K, Denenberg A, Malhotra V, Wong HR (2004). Epigallocatechin-3-gallate, a green tea-derived polyphenol, inhibits IL-1*β*-dependent proinflammatory signal transduction in cultured respiratory epithelial cells. *The Journal of Nutrition*.

[36] Aktas O, Prozorovski T, Smorodchenko A (2004). Green tea epigallocatechin-3-gallate mediates T cellular NF-*κ*B inhibition and exerts neuroprotection in autoimmune encephalomyelitis. *The Journal of Immunology*.

